# Role of Syndecans in Ovarian Cancer: New Diagnostic and Prognostic Biomarkers and Potential Therapeutic Targets

**DOI:** 10.3390/cancers15123125

**Published:** 2023-06-09

**Authors:** Julia Oto, Quang-Khoi Le, Sebastian D. Schäfer, Ludwig Kiesel, Josep Marí-Alexandre, Juan Gilabert-Estellés, Pilar Medina, Martin Götte

**Affiliations:** 1 Department of Gynecology and Obstetrics, Münster University Hospital, 48149 Münster, Germany; juliaotomartinez@gmail.com (J.O.); quang-khoi.le@ukmuenster.de (Q.-K.L.); sebastiandaniel.schaefer@ukmuenster.de (S.D.S.); ludwig.kiesel@ukmuenster.de (L.K.); 2 Haemostasis, Thrombosis, Arteriosclerosis and Vascular Biology Research Group, Medical Research Institute Hospital La Fe, 46026 Valencia, Spain; medina_pil@gva.es; 3 Research Laboratory in Biomarkers in Reproduction, Gynaecology and Obstetrics, Fundación Hospital General Universitario de Valencia, 46014 Valencia, Spain; josepmarialexandre@gmail.com (J.M.-A.); juangilaeste@yahoo.es (J.G.-E.); 4 Department of Pathology, Consorcio Hospital General Universitario de Valencia, 46014 Valencia, Spain; 5 Department of Gynecology and Obstetrics, Consorcio Hospital General Universitario de Valencia, 46014 Valencia, Spain; 6 Department of Paediatrics, Obstetrics and Gynecology, University of Valencia, 46010 Valencia, Spain

**Keywords:** ovarian cancer, syndecan family, syndecan-1, syndecan-2, syndecan-3, syndecan-4, diagnosis biomarker, prognosis biomarker, therapeutic target, proteoglycan

## Abstract

**Simple Summary:**

Ovarian cancer has high prevalence and mortality in women. An early diagnosis of cancer is associated with a better prognosis of the oncologic patients. Ovarian cancer generally presents non-specific symptoms, and thus is frequently diagnosed only when the patients have an advance stage of the disease, resulting in higher mortality. Cancer cells are surrounded by other molecules and cells and the interaction of the cancer cells with these other components plays an essential role in the development of the disease. Syndecans are a family of four transmembrane proteins, which are dysregulated in a myriad of cancers, including ovarian cancer. Many previous studies suggest that these proteins are promising diagnostic and prognostic biomarkers for ovarian cancer. Furthermore, the study of these proteins in ovarian cancer could lead to the discovery of new drugs for the treatment of ovarian cancer.

**Abstract:**

Ovarian cancer (OC) is the eighth cancer both in prevalence and mortality in women and represents the deadliest female reproductive cancer. Due to generally vague symptoms, OC is frequently diagnosed only at a late and advanced stage, resulting in high mortality. The tumor extracellular matrix and cellular matrix receptors play a key role in the pathogenesis of tumor progression. Syndecans are a family of four transmembrane heparan sulfate proteoglycans (PG), including syndecan-1, -2, -3, and -4, which are dysregulated in a myriad of cancers, including OC. Many clinicopathological studies suggest that these proteins are promising diagnostic and prognostic biomarkers for OC. Furthermore, functions of the syndecan family in the regulation of cellular processes make it an interesting pharmacological target for anticancer therapies.

## 1. Introduction

Ovarian cancer (OC) is the eighth cancer both in prevalence and mortality in women, with 314,000 new diagnoses and is responsible for 207,000 deaths in 2021 [1]. OC is the deadliest female reproductive cancer. Frequently, it is not diagnosed until it is at an advanced stage, due to its generally vague symptoms [2]. OC is rare in women under 40 years of age and has 5-year survival rates below 45% overall [3]. OC can be classified into three main histological subtypes. The most common is epithelial, which includes four primary histologic subtypes, namely, the endometrioid, the mucinous, the serous, and the clear cell subtype. Within serous OC, high-grade serous carcinomas (HGSC) are distinguished from low-grade serous carcinomas (LGSC). While HGSCs comprise 70–80% of all epithelial OC subtypes, LGSCs amount to less than 5%. While the endometroid and clear cell subtype show a prevalence of 10%, the mucinous subtype represents only 3% of epithelial OCs. Finally, OCs of sex cord-stromal and germ cell origin represent about 5% of all OCs [2]. 

OC is often asymptomatic in early stages, and thus it is considered a silent killer. While curative therapy is possible in early stages of the disease, this is usually not the case in later stages characterized by extensive intraperitoneal spread. These facts reveal the need for identifying novel early detection biomarkers and new therapeutic targets for advanced stages [4].

One main factor in malignancy is the tumor extracellular matrix (ECM), which plays a key role in the pathogenesis of tumor progression. Indeed, altered expression of ECM macromolecules in the tumor microenvironment (TME) affects virtually all processes during cancer development, such as cancer cell growth, survival, adhesion, and migration [5]. Apart from large structural glycoproteins, such as, e.g., collagens, laminins, fibronectin and tenascins, proteoglycans (PGs) are a major and functionally relevant constituent of the ECM [6]. Proteoglycans are a subclass of glycoproteins to which a linear carbohydrate chain of the glycosaminoglycan (GAG) type is attached. GAGs of PGs are composed of repetitive disaccharide units of uronic acid and an amino sugar, which are substituted with sulfate residues at specific positions, resulting in a highly negatively charged biomolecule that can bind to a multitude of ligands. For example, heparan sulfate is composed of repetitive units of N-acetylglucosamine-α-L-iduronic acid/β-D-glucuronic acid. Based on criteria, such as cellular and subcellular location, sequence homology, and the presence of core protein modules, four major PG classes have been described, distinguishing intracellular, cell-surface, pericellular, and extracellular proteoglycans [7]. 

**Figure 1:**
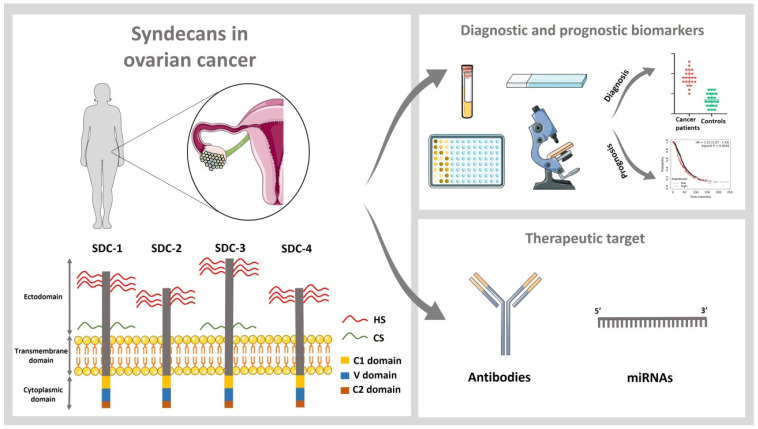
Overview of the value of the syndecan family as diagnostic biomarkers and therapeutic targets in ovarian cancer. SDC1: Syndecan-1; SDC2: Syndecan-2; SDC3: Syndecan-3; SDC4: Syndecan-4; HS: Heparan sulfate; CS: Chondroitin sulfate; C1: Constant domain 1; C2: Constant domain 2; V: Variable domain.

Cell surface heparan sulfate (HS) PGs of the syndecan family play an important role in several disorders, including cancer [8]. Specifically, syndecans are a family of four transmembrane heparan sulfate PGs, including syndecan-1 (SDC1, CD138), -2 (SDC2, HSPG1), -3 (SDC3), and -4 (SDC4) in mammals. Each syndecan is formed by an extracellular domain, a transmembrane domain, and a cytoplasmatic domain (Figure 1). The extracellular domain presents low amino acid sequence homology among the different members of the family and has different attachment sites for carbohydrate chains of the GAG type, namely, HS and, in some cases, chondroitin sulfate (CS) [9]. The transmembrane domain allows for syndecan oligomerization, which can occur in a homotypic or heterotypic manner. The two constant regions (C1 and C2) within the cytoplasmic domain of all syndecans are separated by a variable domain (V), which is specific for each syndecan [10]. 

All members of the syndecan family are dysregulated in a large variety of cancers, including breast [11], pancreatic [12], colorectal [13], prostate [14], among others. Syndecans regulate several cellular processes that are mechanistically related to tumor progression, including cell proliferation, cell migration and invasion, as well as tumor angiogenesis. They act as adhesion receptors and modulate various signaling pathways as co-receptors for receptor tyrosine kinases, G-protein-coupled receptors, and stemness-associated signaling pathways, including the Notch pathway [15]. They have also been proposed as diagnosis prognosis biomarkers and interesting targets for drug development in cancer [10].

In the next sections, we will discuss the role of the syndecan family in OC and the value as biomarkers and therapeutic targets (Figure 1).

## 2. Syndecans as Diagnostic and Prognostic Biomarkers in Ovarian Cancer

In this section, we will review all the studies that have proposed syndecans as diagnostic or prognostic biomarkers of OC (the characteristics of the studies and the main obtained results are summarized in Table 1).

The work of Casey RC et al. [16] showed that proteoglycan transcripts were detected in high amounts in NIH:OVCAR5 cells, including syndecan-4, the secreted dermatan sulfate proteoglycan decorin, the basement membrane proteoglycan perlecan, and the less frequently studied bamacan. Although in lower amounts, these cells also expressed transcripts of cell surface proteoglycans, including the transmembrane proteoglycans syndecan-1 and syndecan-2, the glycosylphosphatidylinositol-anchored glypican-1 and glypican-4, the hyaluronan receptor CD44, secretory granule proteoglycan 1, and the interstitial proteoglycan versican. Several of these proteoglycans have a documented role in ovarian cancer pathogenesis, which has been reviewed elsewhere [17,27,28]. Moreover, the mRNA expression of these proteoglycans was studied in normal human ovaries and omentum, primary OC, and OC metastases in the omentum. However, regarding the syndecan family in human samples, they only found an upregulation in syndecan-1 comparing ovarian carcinoma tumor versus normal ovary and secondary omental metastases versus normal omenta.

Davies E.J. et al. [17] employed immunohistochemistry for all syndecan family members, perlecan, and glypican in normal ovary and pathologically-altered samples ranging from benign lesions over borderline and malignant ovarian cancer. While syndecan-1 staining was absent in normal ovary, it could be detected both in epithelial and stromal cells of the pathologically altered entities. Disruption of basement membranes by cancer cells was associated with a reduction in perlecan staining, while perlecan staining in the vascular basement membranes remained intact. In contrast to syndecan-1, expression of glypican-1 and the remaining three members of the syndecan family was detected in normal, benign, and malignant ovarian tissue. Of note, a prognostic value could be assigned to syndecan-1 and glypican-1, as both correlated with a poor overall survival (OS) and progression-free survival (PFS) of OC patients.

Salani R. et al. [18] validated by RT-qPCR in ovarian tumors the gene expression of 16 ECM proteins found in an in silico analysis. They replicated the upregulation of the syndecan-1 transcript in tumor samples. Similar to the study of Davies EJ et al. [17], immunohistochemical staining for syndecan-1 revealed its upregulation of malignant ovarian tissues compared to benign samples and controls.

Kusumoto T. et al. [19] aimed to study the expression of syndecan-1 and versican in 111 epithelial OC patients. The authors found an opposite effect on the expression of epithelial versus stromal syndecan-1 in OC patients. Epithelial syndecan-1 expression was significantly lower in patients with advanced disease and the PFS rates of patients with epithelial syndecan-1 expression were significantly higher than those of patients with negative epithelial syndecan-1 expression. On the contrary, the stromal syndecan-1 expression was significantly higher in patients with advanced disease. The PFS and OS rates of patients with high stromal syndecan-1 expression were significantly lower than those of patients with low stromal syndecan-1 expression.

Guo Q. et al. [20] studied the syndecan-1 expression (transcript and protein) in normal ovary and ovary tumors. In agreement with the aforementioned studies [17,19], syndecan-1 was not detected in normal ovarian tissue, but expressed in the epithelial compartment of benign lesions, borderline tumors, and ovarian adenocarcinomas. Syndecan-1 expression was present both in the primary tumor and metastases. These findings reinforce the contribution of stromal expression and changes in the expression of syndecan-1 in the pathogenesis and metastasis of OC.

Hasby E.A. [21] analyzed the expression of syndecan-1 in HGSC, LGSC, and clear cell carcinomas and found that its expression was higher in HGSC and clear cell carcinoma compared to LGSC. In addition, syndecan-1 expression correlated significantly to FIGO stage in his study patients. 

Lundgren et al. [22] took the inflammatory microenvironment of epithelial OC into account by evaluating B-cell and plasma cell infiltration via immunohistochemistry for immunoglobulin kappa C, CD20, and syndecan-1. Analyzing over 150 cases, a significant correlation between IGKC and CD20 as well as syndecan-1 was revealed. Both syndecan-1 and CD20 expression were correlated with tumor grade. Finally, univariate Cox regression analysis revealed an inverse correlation between SDC1 expression and OS as well as OC-specific survival.

Klaschik S. et al. [23] analyzed the concentration of different molecules in pre- and post-operative cytoreductive surgery serum samples of 26 late-stage OC patients, including syndecan-1, using multiplex protein arrays and ELISAs. Syndecan-1 was significantly increased in the post-surgery samples compared to baseline conditions.

Kulbe H. et al. [24] identified promising novel candidates for epithelial OC, including syndecan-3, and found the stroma of the tumor to be a suitable compartment for biomarker discovery. First, they performed an in silico analysis using three datasets from the gene expression omnibus (GEO) database, which contains the information of expression profiling of OC related genes: GSE29156, GSE40595, and GSE14407. Next, they selected a series of dysregulated mRNAs as biomarker candidates and designed a custom mRNA set of Versican (VCAN), syndecan-3, and aurora kinase A (AURKA). In addition, T-cell differentiation protein myelin and lymphocyte (MAL) were confirmed as potential biomarkers for epithelial OC.

Kim S. et al. [25] were able to distinguish seven distinct cell types by means of single-cell RNA sequencing of ascites cells isolated from five OC patients. The authors demonstrated that the chemokine CCL5 was enriched in immune cells, and speculated that syndecan-4 may be involved in mediating its impact on improved OC cell survival in ascites. Finally, performing an in silico analysis with public databases from TCGA and GEO (cohorts GSE9891, GSE3149, GSE26193, and GSE63885), the authors found that syndecan-1 expression level negatively correlated with OS in OC patients and that syndecan-4 expression increased across tumor stage.

Considering an in silico approach, Li X. et al. [26] analyzed three datasets (GSE38666, GSE40595, and GSE66957) from the GEO database. Hub genes were analyzed by GO, Kyoto Encyclopedia of Genes, and protein expression studies, revealing close to 200 differentially expressed genes. Survival analysis of SDC1, COL4A1, CDKN2A, and TOP2A as hub genes revealed that they were correlated with OC patient OS. Expression of the hub genes could be independently validated using the Human Protein Atlas and the GEPIA database.

In addition to the abovementioned studies, we performed an in silico analysis of mRNA expression of the syndecan family members in OC patients employing the online survival analysis tool KMplot [29], which combines gene expression datasets of more than 1600 patients. Details on the patient collective can be found in the original reference [29]. Gene expression data from tumor samples of OC patients were analyzed by stratifying patients according to high and low expression of syndecan using the default settings of the KM Plotter tool. Syndecan expression was studied in relation to OS, PFS, and post-progression survival (PPS).

**Figure 2:**
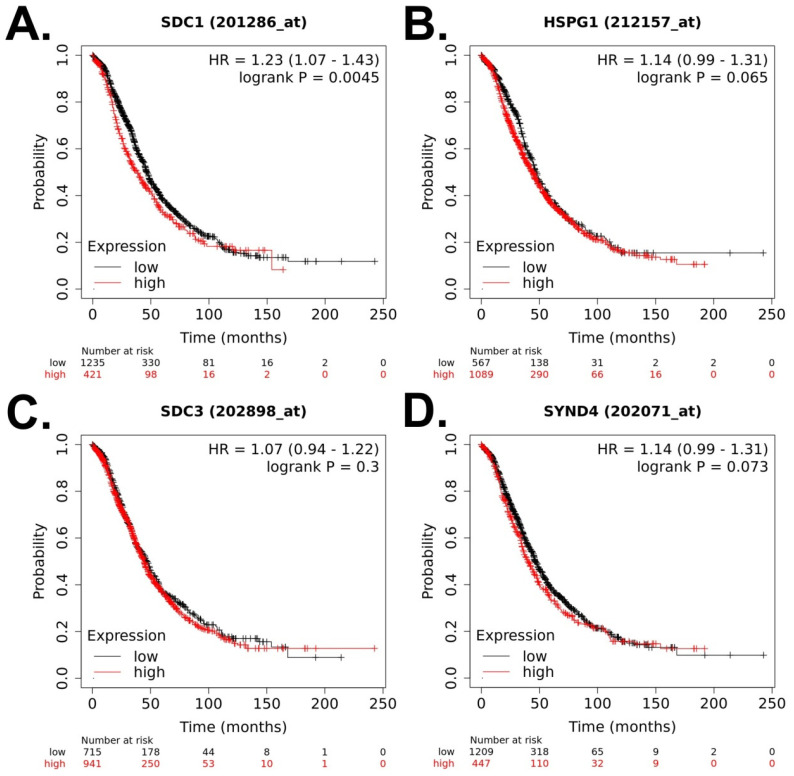
Prognostic value of syndecan family members expression for overall survival of patients with ovarian cancer based on the dataset of the Kaplan–Meier plotter [29] (data retrieved on 15 March 2023). The default settings in the KM Plotter tool were used to calculate the overall survival. (**A**) SDC1 (syndecan-1), (**B**) SDC2 (HSPG1, syndecan-2), (**C**) SDC3 (syndecan-3), (**D**) SDC4 (syndecan-4).

**Figure 3:**
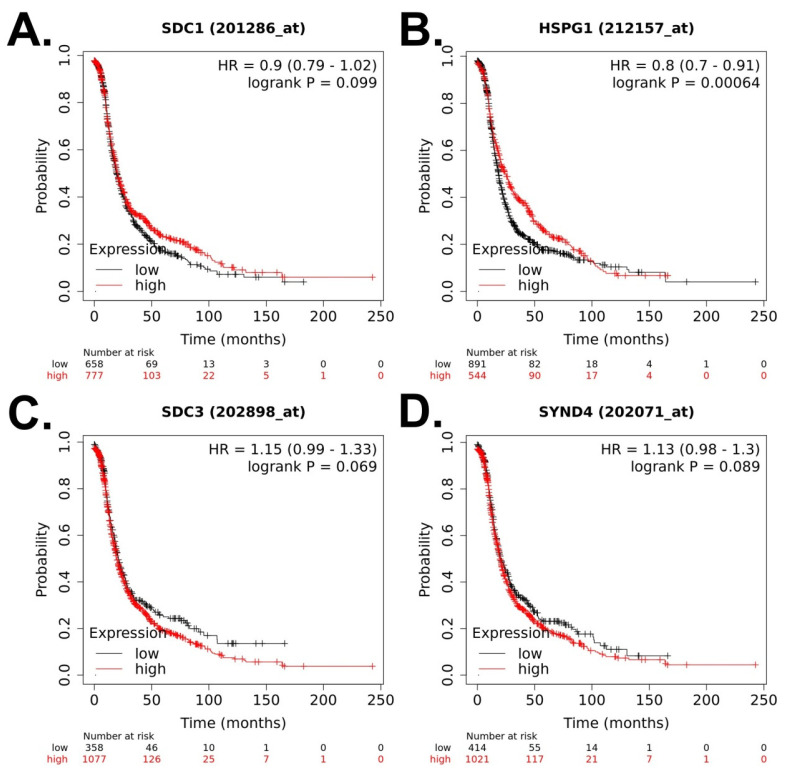
Prognostic value of syndecan family member expression for progression-free survival of patients with ovarian cancer based on the Kaplan–Meier plotter [29] (data retrieved on 15 March 2023). The default settings in the KM Plotter tool were used to calculate the progression-free survival. (**A**) SDC1 (syndecan-1), (**B**) SDC2 (HSPG1, syndecan-2), (**C**) SDC3 (syndecan-3), (**D**) SDC4 (syndecan-4).

**Figure 4:**
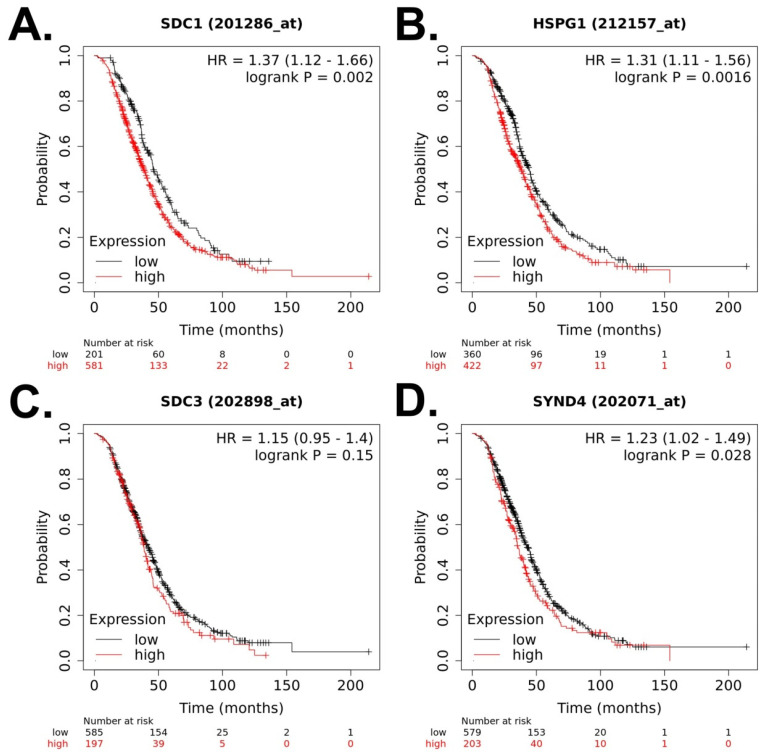
Prognostic value of syndecan family member expression for post-progression survival of patients with ovarian cancer based on the Kaplan–Meier plotter [29] (data retrieved on 15 March 2023). The default settings in the KM Plotter tool were used to calculate the post-progression survival. (**A**) SDC1 (syndecan-1), (**B**) SDC2 (HSPG1, syndecan-2), (**C**) SDC3 (syndecan-3), (**D**) SDC4 (syndecan-4).

First, we analyzed the prognostic value of syndecan-1, -2, -3, and -4 expression for OS in OC patients (N = 1657) (Figure 2A–D). Higher expression of syndecan-1 was related to lower OS (hazard ratio, HR = 1.23, 95% confidence interval (CI = 1.07–1.43), *p* = 0.0045) (Figure 2A). Next, we analyzed the prognostic value for PFS in OC patients (N = 1436) (Figure 3A–D). Higher expression of syndecan-2 was related to higher PFS (HR = 0.8, 95% CI (0.7–0.91), *p* = 0.0006) (Figure 3B). Finally, we analyzed the prognostic value of these molecules for PPS in OC patients (N = 782) (Figure 4A–D). Higher expression of syndecan-1, -2, and -4 was related to lower PPS: Syndecan-1 HR = 1.37 (95% CI (1.12–1.66), *p* = 0.002, Figure 4A), syndecan-2 HR = 1.31 (95% CI (1.11–1.56), *p* = 0.0016, Figure 4B), syndecan-4 HR = 1.23 (95% CI (1.02–1.49), *p* = 0.028, Figure 4D).

Overall, these studies provide evidence for the dysregulation of individual syndecan family members in OC, and suggest their relevance for disease prognosis.

## 3. Pathophysiological Role of Syndecans in Ovarian Cancer Tumorigenesis

The different members of the syndecan family have been previously related to tumorigenesis at the mechanistic level in various tumor entities [10,15]. Here, we focus on the role of these molecules in OC biology. Regarding the function of syndecans, it has to be considered that an essential functional element of their extracellular domains is the GAG chain of the heparan sulfate (and occasionally chondroitin sulfate) type. These highly negatively charged unbranched GAGs are capable of interacting with numerous ligands relevant to tumor progression under physiological conditions, including growth factors, chemokines and morphogens and their receptors, ECM constituents, proteases and protease inhibitors, and factors involved in the regulation of haemostasis [10,30,31]. While the technical analysis of specific functions of syndecans in OC is in most cases linked to the manipulation of core protein expression, it should be considered that syndecan function is strongly influenced by the fine structure of the glycosaminoglycan chain. For example, the HS 3-O-Sulfotransferase HS3ST2 is epigenetically silenced in OC, and related to a poor prognosis in epithelial OC. Moreover, overexpression of HS3ST2 in the OC cell line SKOV3 resulted in reduced invasion, migration, and a reduction in IL-6-mediated STAT3-activation [32]. Furthermore, an important role for 6-O-sulfation for OC angiogenesis in vitro and in vivo was demonstrated employing an shRNA-mediated knockdown approach of the HS 6-O-sulfotransferases HS6ST1 and HS6ST2 in human ovarian cancer cell lines [33]. Reduction in 6-O-sulfation of HS linked to the downregulation of the biosynthetic enzymes resulted in diminished EGFR activation and associated downstream signaling, reduced angiogenesis in vitro, and a delay of OC xenograft growth in vivo, highlighting the role of specific HS sulfation patterns for disease progression. The role for 6-O-sulfated HS was also highlighted in a number of studies on the HS editing enzyme HSulf-1, which selectively cleaves 6-O-sulfate from HS in OC. Polymorphisms in the HSulf-1 gene were associated with early onset and survival in OC [34], while at the functional level, 6-O-sulfate editing by HSulf-1 has been linked to reduced heparin-binding growth factor signaling, including VEGF, FGF-2, and HB-EGF, reduced tumor cell proliferation, and enhanced sensitivity to chemotherapy in vitro [35,36]. Moreover, the HS degrading enzyme heparanase is overexpressed in OC and associated with a poor prognosis [37]. At the functional level, heparanase enhanced proliferation and invasive growth of OC cells in vitro and in vivo [38]. While these data emphasize the important role for HS as a major functional domain in the syndecans of OC, it has to be considered that manipulation of HS also affects additional proteoglycans. Therefore, these data cannot be fully extrapolated to syndecan function.

Regarding the syndecans, early studies in OC cell lines (SKOV3, NIH:OVCAR3) showed that adhesion to interstitial matrix molecules (collagen I, collagen III, and fibronectin) can be competitively inhibited by heparin, heparan sulfate, and chondroitin/dermatan sulfate, and by enzymatic degradation of cell surface heparan sulfate and chondroitin sulfate [39]. PCR analysis confirmed expression of syndecan-1 and syndecan-2 in cellular models, suggesting a possible contribution of these cell surface proteoglycans to this process.

Matsuzaki H. el al. studied the effect of syndecan-1 inhibition in the human OC cell line HRA [40]. A reduced proliferation was evidenced in those cells transfected with antisense syndecan-1 oligodeoxynucleotide and, therefore, with lower levels of syndecan-1. This observation is in accordance with the classical co-receptor role of syndecan-1 for growth factor signaling; however, while the proliferative response to the cytokines HB-EGF, HGF, or FGF-2 was reduced upon syndecan-1 downregulation, the response to IGF-1 remained the same as in controls, indicating cytokine-specific effects. Surprisingly, the transfected cells had an increased heparin-binding growth factor-dependent invasiveness. While we can currently only speculate on the mechanisms, this finding parallels observations in syndecan-1-depleted breast cancer cells, which show a reduced response to FGF-2-mediated signaling [41], but enhanced activation of focal adhesion kinase (i.e., integrin-like signaling) and enhanced invasiveness [42].

Indeed, the role of cell surface HS proteoglycans as co-receptors for receptor tyrosine kinase signaling mediated by the cytokine fibroblast growth factor-2 (FGF-2) is well-documented [9,15]. Whitworth M.K. et al. [43] studied the role of the potent angiogenic cytokine fibroblast growth factor-2 (FGF-2) in OC, which is dependent on HS for its biological activity. They aimed to investigate the relationship among HS, FGF-2, and the signal-transducing receptors in HGSC. In ovarian serous adenocarcinoma tissue, the subset of FGF-2-binding HS that is capable of activating FGF-2 was expressed predominantly by endothelial cells as well as the stroma. Double staining techniques, for syndecan-3 and CD31 assessed the expression of syndecan-3 expression in tumor blood vessels. Moreover, using double staining, HS was predominantly found at the basal aspect of the endothelium, suggesting that syndecan-3 might function as one of the proteoglycans involved in FGF-2 signaling in the endothelium.

Tumbarello D.A. et al. [44] studied how components of the ECM are implicated in the response to paclitaxel-induced cell death in OC cells. Previously, they demonstrated that integrin-related signaling pathways, including FAK and the GTPase RhoA are mechanistically involved in sensitizing OC cells to paclitaxel by promoting microtubuli stabilization. The secreted ECM protein transforming growth factor beta induced (TGFBI) was involved in activating this pathway, demonstrating the relevance of the tumor microenvironment for therapeutic responses in OC [45]. Notably, siRNA-mediated silencing of syndecan-1 expression was demonstrated to act synergistically with sh-RNA-depletion of ß1 integrin in SKOV3 OC cells, resulting in increased adhesion to TGFBI [44], and thus provides a link between syndecan-1 function and chemoresistance in OC. 

Kato N. et al. [46] aimed to study the inflammatory component of the stroma in ovarian cell carcinoma. Only 20% of the cases (12 out 60 of the studied tissues) presented an inflammatory stroma. Interestingly, the main infiltrated cells in the stroma were CD138 (syndecan-1-)-positive differentiated plasma cells. While syndecan-1 was mainly used as a marker for plasma cells in this study, its presence may indicate a possible role in antibody-mediated antitumoral responses in a subset of OC patients.

Ding Y. et al. [47] identified an elegant molecular mechanism linking syndecan-3 function to the progression of OC. Using fallopian tube tissue as a control, the authors demonstrated that expression of the long non-coding RNA TRPM2-AS and of syndecan-3 were upregulated in OC tissue, whereas expression of the microRNA miR-138-5p, predicted to target syndecan-3 expression, was downregulated in the tumors. microRNAs are small noncoding RNAs which regulate gene expression at the posttranscriptional level, and which are frequently dysregulated in cancer. Notably, several microRNAs were demonstrated to modulate pro- or anti-tumorigenic effects by targeting proteoglycan expression [48]. In an elegant set of in vitro experiments, the authors demonstrated that TRPM2-AS acts as a sponge for miR-138-5p, thus releasing syndecan-3 from inhibition [47]. As a consequence, both TRPM2-AS and syndecan-3 promoted several pathophysiological processes related to OC progression in vitro, including colony formation, OC cell migration, and invasion. Moreover, TRPM2-AS downregulation inhibited xenograft tumor growth in vivo and acted synergistically with cisplatin chemotherapy in vitro.

Hillemeyer L et al. [49] utilized in silico analysis using the online tool TNMplot to reveal a significant upregulation of syndecan-3 mRNA in tumor tissue and metastases compared with normal tissue. At the functional level, syndecan-3 depletion impaired 3D spheroid growth and colony formation as stemness-related readouts in SKOV3 and CAOV3 cells. These phenotypic changes were associated with reduced STAT3 signaling in line with a classical co-receptor function for syndecan-3, and a transcriptional dysregulation of several constituents of the stemness-associated Notch, WNT, and hedgehog signaling pathways. These findings point at a possible role for syndecan-3 in cancer stem cells, or tumor-initiating cells, which are discussed to represent a cell pool characterized by increased resistance to conventional therapies due to the expression of multidrug resistance proteins, increased DNA repair capacity, high developmental plasticity, and unlimited proliferation [50]. 

The benign disease endometriosis is associated with an increased risk of OC, mainly in endometrioid and clear cell subtypes [51]. Investigating molecular mechanisms associated with ovarian endometriosis, Ponandai-Srinivasan et al. could demonstrate that silencing syndecan-1 expression in 3D endometriotic cell spheroids resulted in a downregulation of cancer-associated pathways, including WNT and G-protein coupled receptor signaling, as revealed by transcriptomics [52]. Along with the observation that 3D endometriotic cells spheroids exhibiting high expression of syndecan-1 showed increased invasive growth in the presence of TGFbeta, these data may provide a possible mechanistic link of aberrant syndecan-1 expression to the pathogenesis of endometriosis-associated OC.

Apart from the examples for a mechanistic contribution of individual syndecans to OC pathogenesis mentioned above, some aspects of syndecan function remain to be studied in this context. For example, syndecan-1 has been mechanistically linked to the biogenesis of exosomes, membrane-bound extracellular vesicles derived from the endosomal compartment, forming a complex with the cytoplasmic adaptor protein syntenin and ALIX [5,10,53]. This is of pathophysiological relevance in an oncological context, such as, e.g., in myeloma, chemotherapy can induce secretion of so-called chemoexosomes which deliver cargo, including the HS degrading enzyme heparanase to myeloma cells. This process results in the shedding of cell surface syndecan-1 and is discussed to enhance chemoresistance and patient relapse [54]. While speculative at this point, similar mechanisms may be of relevance and worth addressing in OC, as exosomes have been proposed as diagnostic markers for disease progression and as potential therapeutic targets [55,56]. At the molecular level, exosomes transfer pro-tumorigenic cargo to the tumor microenvironment promoting OC pathogenesis by promoting conversion of fibroblasts to cancer-associated fibroblasts, facilitating immune-escape mechanisms and by remodeling of the mesothelium to enhance peritoneal dissemination [55,56]. Therefore, altered syndecan-1 function in OC may additionally contribute to these processes at the level of exosome formation.

## 4. Syndecans as Therapeutic Targets in Ovarian Cancer

The dysregulation of the different members of syndecan family in multitude of cancers, and its role in different steps of tumor progression and invasiveness, postulate these molecules as potential targets for cancer therapy. Subsequently, we review different studies in which syndecans have been targeted as therapy in OC.

Helpman L. et al. [57] employed flow cytometry to evaluate the expression of syndecan-1 and 29 additional hematologic antigens on human OC cell lines, and to study a possible impact of estrogen on their expression patterns. These analysis were independently evaluated on OC tissues by immunohistochemistry. While no estrogen-dependent expression was observed, the authors concluded that the studied antigens may serve as putative drug targets due to their cell surface accessibility. However, while this is indeed a point of consideration for druggable targets [58] as a caveat, it has to be considered that the targets may also be expressed on healthy tissue, and that further characterization is needed to fully validate the suitability of these antigens as therapeutic targets in OC.

Guo T. et al. [59] studied the role of miR-302a as an epigenetic regulator of syndecan-1 in OC. In vitro reporter assays suggested that syndecan-1 is a direct target gene of miR-302a. The authors showed that the expressions of miR-302a in OC cells were inversely correlated with those of syndecan-1 and that the upregulation of syndecan-1 could rescue the effect of miR-302a overexpression in the OC cells, which included an inhibition of OC cell proliferation and an increase in apoptosis. The authors suggest that exogenous miR-302a could be a potential therapeutic target in OC thanks to its role as a regulator of syndecan-1.

The group of Carnemolla B has extensively studied the use of anti-syndecan-1 antibodies as a therapeutical strategy in different tumors. Using the human recombinant antibody OC-46F2, which recognizes the extracellular domain of syndecan-1, they demonstrated therapeutic efficacy in an in vivo SCID mouse xenograft model of melanoma and OC cell growth [60]. A targeting of tumor vessel maturation in the angiogenic microenvironment was identified as part of the molecular mechanism in this context. Later, they ascertained the use of the OC-46F2 recombinant antibody in combination with the tumor-targeted antibody-based immunocytokine L19-IL2, which targets a fibronectin isoform enriched in tumor neovasculature, for melanoma therapy. Combinatorial targeting of syndecan-1 and B-fibronectin with these reagents resulted in a synergistic effect and more efficient inhibition of tumor growth in vivo in a mouse model [61]. In 2019, they published the study of this combined therapy for OC treatment, confirming the increased efficacy of the combined therapy compared to monotherapy. Moreover, they noted a significant increase in syndecan-1 levels in the serum of OC patients compared to healthy donors, and increased syndecan-1 levels in more advanced stages of OC. Mechanistically, combinatorial treatment resulted in altered EMT marker expression and a reduction in stemness-associated properties of the targeted xenografted SKOV3 OC cells, in addition to a reduction in vascular mimicry, which is in accordance with the previous observations in the melanoma model [62].

Carvalho R.F. et al. [63] used RNA-sequencing to identify relevant ligands and receptors mediating the communication between cancer-associated fibroblasts and serous OC cells and organoids. They identified as potential therapeutic target the ligand-receptor interaction between midkine (MDK, expressed in cancer-associated fibroblasts), a growth factor that acts on cancer progression and NCL/SDC2/SDC4 (expressed in cancer cells), highlighting the co-receptor role of syndecans for pro-tumorigenic signaling. In contrast, investigations of the specific role of syndecan-3 in MDK signaling via STAT3 in OC cell lines were inconclusive, as syndecan-3 depletion reduced STAT3 signaling independent of midkine stimulation [49]. 

Finally, syndecans may also indirectly modulate the response of OC cells to conventional therapies. For example, in EOC, Decoy Receptor 3 (DcR3), a soluble protein member of the tumor necrosis factor receptor family, has been linked to platinum resistance [64]. Employing enzymatic digestion of cell surface heparan sulfate on OC cells in vitro, Connor et al. demonstrated that DcR3 binds to heparan sulfate, and that this process is linked to platinum resistance [65]. Syndecan-2 and CD44v3 were identified as major HS proteoglycans in the in vitro model suggesting a possible involvement of syndecan-2 in therapeutic resistance.

## 5. Conclusions

OC is the deadliest female reproductive cancer. High mortality is partly due to the fact that OC diagnosis is belated to advanced stages, due to its generally unspecific symptoms. All members of the syndecan family are dysregulated in a large variety of cancers, including OC. Many studies have proven these proteins as promising diagnostic and prognostic biomarkers and novel therapeutic targets for OC. This is supported by the findings of our KM Plotter analysis, which assigns a negative prognostic value for syndecan-1 regarding OS, and for syndecan-1, -2, and -4 for PPS. In contrast, no prognostic value was assigned to syndecan-3, suggesting a syndecan-specific impact on survival in OC. Surprisingly, high expression of syndecan-2 was associated with better PFS, while it is associated with poor PPS. This finding may suggest a dual role for this proteoglycan, which may act on different pathologic processes during tumor progression, which play distinct roles regarding PFS and PPS. Validation in larger independent cohorts of patients and controls is needed if these findings ought to be transferred to clinical practice. With respect to OC pathophysiology, several syndecan family members fulfil functions according to the classical co-receptor role of these proteoglycans for growth factor signaling, including FGF-2, HB-EGF, HGF, TGFbeta, and MDK. These signaling roles are complemented by data indicating cooperation with integrin-related pathways, which need to be explored in more detail in future studies. Apart from regulating OC cell proliferation, migration and invasion, a novel role for syndecans in cancer stem cell function and their epigenetic regulation by noncoding RNAs are emerging topics in the field worthy of further exploration. Due to their mechanistic and prognostic relevance, the syndecans have emerged as drug targets in OC, with preclinical studies showing encouraging results.

## Figures and Tables

**Table 1:** Studies evaluating syndecan-1, -2, -3, and -4 as biomarkers in ovarian cancer patients.

N	Type of Sample	Detection	Relevant Finding	Reference
50 control ovaries 20 primary OCs 17 omental metastases 7 normal omenta	Frozen tissue	Gene expression	SDC1 upregulated in ovarian carcinoma tumor vs. normal ovary and in secondary omental metastases vs. normal omenta.	Casey RC et al., 2003 [16]
115 EOC patients 10 benign epithelial ovarian tumor patients 12 controls	FFPE tissue	IHC	SDC2, -3, and -4 were expressed in both normal ovary and benign and malignant ovarian tumors. Negative expression of SDC1 in controls, positive in tumors. Presence of stromal SDC1 and its intensity were associated with poor OS and PFS.	Davies EJ et al., 2004 [17]
138 EOC patients 17 atypical proliferative serous tumors 22 ovarian serous cystadenomas 12 controls	FFPE tissue	RT-qPCR IHC	SCD1 expression (mRNA and protein) upregulated in OC samples.	Salani R. et al., 2007 [18]
111 patients	FFPE tissue	IHC	Epithelial SDC1: Lower expression in patients with advanced disease. Higher PFS in patients with negative expression. Stromal SDC1: Higher expression in patients with advanced disease. Lower PFS and OS in patients with high expression compared to patients with low expression.	Kusumoto T et al., 2010 [19]
26 EOC patients 5 borderline 27 benign 2 controls	FFPE tissue	RT-qPCR IHC	Negative expression of SDC1 in controls, positive in tumors, and in borderline samples. Expression of both syndecan-1 and its mRNA detected at the original site of the tumor and in the metastatic foci.	Guo Q et al., 2015 [20]
41 EOC patients	FFPE tissue	IHC	SDC1 expression higher in HGSC and clear cell carcinoma compared to LGSC. SDC1 expression correlated significantly to FIGO stage.	Hasby E.A. 2016 [21]
154 EOC patients 38 benign 33 omental metastases	FFPE tissue (TMAs)	IHC	High CD20 and SDC1 expression correlated significantly with high tumor grade. High SDC1 expression correlates with a poor OS and with poor ovarian cancer-specific survival.	Lundgren et al., 2016 [22]
26 late-stage ovarian cancer patients (pre- and post-cytoreductive surgery)	Serum	Multiplex array	SDC1 was significantly increased in the post-surgery samples compared to baseline conditions.	Klaschik S et al., 2019 [23]
Screening: 3 datasets from GEO database (GSE29156, GSE40595, and GSE14407) Validation: 10 EOC patients 10 benign	Tissue Blood	Screening: In silico analysis Validation: Gene expression analysis (custom designed mRNA set containing 48 genes)	SDC3 was confirmed as potential biomarker.	Kulbe H et al., 2019 [24]
Datasets from TCGA and GEO repositories (GSE9891, GSE3149, GSE26193, and GSE63885)	Tissue	In silico analysis	Higher expression of SDC4 is correlated with poor OS. SDC4 expression increased across the tumor stage.	Kim S et al., 2021 [25]
3 datasets from GEO database (GSE38666, GSE40595, and GSE66957)	Tissue	In silico analysis (expression profiling, bioinformatic analysis)	Higher expression of SDC1 is correlated with poor OS.	Li X et al., 2022 [26]

EOC: Epithelial ovarian cancer; FFPE: Formalin-Fixed Paraffin-Embedded; FIGO: International Federation of Gynaecology and Obstetrics; HGSC: High-grade serous carcinomas; IHC: Immunohistochemistry; LGSC: Low-grade serous carcinomas; mRNA: Messenger-RNA; PFS: Progression-free survival; OS: Overall survival; RT-qPCR: Reverse transcription polymerase chain reaction; SDC1: Syndecan-1; SDC2: Syndecan-2; SDC3: Syndecan-3; SDC4: Syndecan-4; TMA: Tissue microarrays.
